# Effects of Face-to-Face and Telephone-Based Family-Oriented Education on Self-Care Behavior and Patient Outcomes in Type 2 Diabetes: A Randomized Controlled Trial

**DOI:** 10.1155/2017/8404328

**Published:** 2017-11-22

**Authors:** Masumeh Hemmati Maslakpak, Somaieh Razmara, Zahra Niazkhani

**Affiliations:** ^1^Maternal and Childhood Obesity Research Center, Urmia University of Medical Sciences, Urmia, Iran; ^2^Department of Medical Surgical Nursing, Urmia University of Medical Sciences, Urmia, Iran; ^3^Student Research Committee, Urmia University of Medical Sciences, Urmia, Iran; ^4^Nursing Department, Nursing and Midwifery Faculty, Urmia University of Medical Sciences, Urmia, Iran; ^5^Department of Health Information Technology, Urmia University of Medical Sciences, Urmia, Iran; ^6^Nephrology and Kidney Transplant Research Center, Urmia University of Medical Sciences, Urmia, Iran

## Abstract

**Background:**

Education of patients and their families is the cornerstone of effective diabetes care. The present study aimed to compare the effects of a face-to-face and telephone-based family-oriented educational program on self-care behavior and patient outcomes in type 2 diabetes patients.

**Methods:**

In the present randomized controlled trial, 90 type 2 diabetes patients were randomly divided into three groups of 30 participants: a face-to-face education group, a telephone-based education group, and a control group. The educational program lasted for 3 months. Outcomes evaluated included self-care, fasting blood sugar, hemoglobin A1c, cholesterol, and triglyceride.

**Results:**

The overall self-care scores in the intervention groups were significantly higher than that in the control group (*P* = 0.0001). In addition, lipid profiles significantly improved in the interventions compared to the control (*P* < 0.05). Comparing the two interventions showed better results for the face-to-face group regarding dietary adherence and physical activity, but the latter group had comparable results in blood glucose monitoring, foot care, and cholesterol level.

**Conclusions:**

This study shows the beneficiary effects of a family-oriented education on self-care and patient outcomes. It also shows the potential value of low-cost telephone technology in delivering effective diabetes care.

## 1. Introduction

Due to population ageing and sedentary lifestyle, the prevalence of type 2 diabetes is rapidly growing worldwide with estimated 366 million patients by 2030 [1]. Among various treatment strategies, self-care is considered the cornerstone of care in both developed and developing countries [2]. An effective self-care resulting from self-management can help not only in improving health outcomes but also in reducing costs [3].

Self-care in diabetes consists of knowledge and awareness of methods to control the chronic disease or its complications for patients or their family members in a social context [4, 5]. Self-care can dramatically change the progression and development of diabetes [6]. Thus, multiple educational and counseling self-care or self-management interventions have been developed for diabetic patients. These interventions can play a pivotal role in behavioral change of patients [7]. Face-to-face education is one of the most common educational methods in health care systems. It enables patients to ask questions or discuss their concerns with nurses and also enables nurses to modify incorrect information of patients and to build a dynamic relationship with patients. However, the overcrowded outpatient clinics' atmosphere makes this time-consuming education impossible [8]. Therefore, other practical and economical approaches have been suggested such as telecare services that provide patients with diabetes management services from the convenience of their homes [9]. Telecare enables patients' monitoring and education, data collection, nursing interventions, pain management, and family support through the low-cost technology of telenursing without time and distance barriers [10, 11].

Studies have shown that family support is a major component of a successful self-care [12]. In fact, involving patients' family members beside patients can improve self-care and quality of life of diabetic patients [13, 14]. This is especially true in adults with poor controlled type 2 diabetes mellitus (T2DM) [15]. Family members' involvement in care can also result in enhanced adherence to care protocols [16, 17] as well as in better diabetes control [18, 19]. Most studies have focused on face-to-face family-oriented interventions [20], and few have investigated telephone-based educational programs [21]. To our knowledge, no study has so far compared the efficacy of face-to-face and telephone-based family-oriented education to determine a more effective and feasible protocol. Therefore, the present study aimed to study and compare the effectiveness of family-oriented education delivered via two methods face-to-face and telephone-based methods on self-care behavior and patient outcomes in Iranian patients with T2DM through a randomized controlled trial.

## 2. Methods

### 2.1. Study Population and Design

The present controlled randomized clinical trial (RCT) was conducted at the Urmia Diabetes Association in Iran from 22 December 2014 to 21 March 2015. The Institutional Review Board (IRB) of the Urmia University of Medical Sciences and the Urmia Diabetes Association approved the study protocol. The study was carried out in accordance with the ethical considerations of the World Medical Association (Declaration of Helsinki). All participants gave consent after being informed about the study details. The protocol of this trial was registered in the Iranian Registry of Clinical Trials (registration number IRCT2014112620106N1).

Patients with T2DM, registered at the Urmia Diabetes Association, were included in the trial in the case they met the following criteria: (1) patients having a known history of type 2 (noninsulin requiring) diabetes confirmed by a specialist; (2) patients aged between 18 and 55; (3) patients having no underlying health problems such as a history of psychological disorders, uncontrolled hypertension (defined as resting systolic blood pressure (BP) >180 mmHg or diastolic BP > 110 mmHg), chronic kidney failure, or cardiovascular disease; and (4) patients and their family members having reading and writing literacy. Exclusion criteria consisted of (1) patients' or family members' failure to participate regularly in the educational sessions and (2) unwillingness to participate further in the study. All medication alterations were based on clinical indications.

According to the inclusion and exclusion criteria, there were 250 eligible patients at the Urmia Diabetes Association. The sample size was calculated at 30 patients for each group, resulting in a total of 90 patients, considering a similar study by Jalilian et al. [22], reporting mean and standard deviation of the self-management score in the intervention group at 6.9 ± 1.37 and in the control group at 5.33 ± 1.67 and taking into account the power of 80% and confidence of 95%. Using a random number table, 90 patients were randomly selected among 250 eligible patients and invited for a meeting with research members, where the study objectives were explained, participants' telephone numbers were recorded, and patients' height and weight were measured for the calculation of the body mass index (BMI). The invited participants were asked to bring the results of their last laboratory investigations, and then, the serum levels of HbA1c, fasting blood sugar (FBS), cholesterol, and triglyceride could be recorded from their previous laboratory tests. These lab tests are routinely performed (free of charge) by the Urmia Diabetes Association for all the registered patients twice a month. In the introductory meeting, the participants were also invited to complete the demographic questionnaire, as well as the Summary of Diabetes Self-Care Activities (SDSCA) measure [23].

In the next step, the 90 selected patients were randomly allocated into three equal groups of thirty members, using random allocation software (RAS); the result of which was concealed from the research team and participants: group 1 received face-to-face family-oriented education, group 2 received telephone-based family-oriented education, and group 3 which was the control group received the usual education that included a monthly training class and educational pamphlet. The patients in the experimental groups and one of their family members (a parent, child, or spouse) were invited to attend the educational meeting.

### 2.2. The Educational Intervention

The educational session included appropriate diet and exercise, blood glucose monitoring, foot ulcer prevention, and adherence to medication. Diet education included subjects such as healthy fats, fruits and vegetables, high-fiber cereals and breads, fish and shellfish, and high-quality protein. A ten-point education specific to diabetic foot care, given to prevent foot ulcers, consisted of footwear use for outdoors, footwear use for indoors, washing and drying of feet daily, healthy nail trimming, daily foot inspection, daily footwear inspection, toe space examination, oil/moisturizer use, change of footwear when damaged/ill fitting, and comfortable fit of footwear. With empowerment-based diabetes patient education, the first two authors listened to patients' concerns and engaged them in collaborative problem solving. For example, patients and families concerned about the cost of nutritious foods; therefore, researchers provided information about heart-healthy, portion-controlled, carbohydrate-consistent food choices, high- and low-glycemic index (GI) foods, and basic carbohydrate counting. Patients were recommended to aerobic exercises (walking, cycling, and swimming). These exercises increase the heart rate for a sustained period of time and often are the best choice for diabetics. We recommended aiming for 30 minutes of moderate-to-vigorous intensity aerobic exercise at least 5 days a week or a total of 150 minutes per week. Patients were instructed to write down their blood glucose levels or use blood glucose meters with memory features and bring them to follow-up appointments. In this study, the same educational content was used to educate the two experimental groups via different delivery methods.

In the face-to-face family-oriented education group, the subjects were subsequently divided into 3 smaller groups to attend the educational classes held at the Urmia Diabetes Association, for 3 months: twice a week in the first month and once a week in the second and third months by the first two authors. The timing of these face-to-face classes was between 8 A.M. and 2 P.M. from Saturday to Wednesday. Patients and their designated family members (one fixed member for each patient) chose the appropriate time according to their convenience of attendance. These classes lasted for 20–30 minutes.

In the telephone-based family-oriented education group, the first two authors performed the educational session and the patient and the family member had similar chance to be educated by one of the two research team instructors. The content of the telephone calls included checking the patient's adherence to diet and exercise, blood glucose monitoring, foot ulcer prevention, and adherence to medications, and in case any nonadherence was detected, the instructor tried to analyze the patient's source of problem through interviewing the patient and the family member and suggest solutions for his/her problem in another call. After the patients and their family members (one fixed member) were consulted, the time of the telephone call was set at 9 A.M. to 10 P.M., lasting for 15 to 30 minutes, twice a week in the first and second months and once a week in the third month. Each patient and her/his family member were called separately. During the telephone calls, all questions were also answered. In case the patient and her/his family member did not answer the phone, his/her cell phone was dialed.

After the clinical trial was finished, the control group received the paper-based educational materials of the teaching sessions. At the end of interventions, all participants refilled the SDSCA questionnaire.

### 2.3. The Primary Outcome Measures

The primary outcome measure was the self-care scores and nonadherence or noncompliance to the prescribed regimen, which was assessed by the SDSCA measure. The Persian version of the SDSCA questionnaire, used in the present study, has been validated by Hemmati Maslakpak et al. via forward-backward translation with Cronbach's alpha of 0.80 [24]. The SDSCA measure consists of 17 items addressing 5 domains of self-care: dietary adherence (items 1 to 5), exercising (items 6 and 7), blood glucose monitoring (items 8 and 9), foot care (items 10 to 14), and medication adherence (items 15 to 17); each item scored from 0 to 7. The scores range between 0 (indicating that the patient has not performed any self-care activities over the last seven days) and 7 (indicating that the patient has performed self-care activities on every single day over the last seven days). Higher scores indicate better self-care performance. The total self-care score is calculated by the sum of the items' scores that range between 0 and 119.

In the questionnaire, there was also a section for demographics including age, gender, marital status, number of children, level of education, place of residence, employment status, income status, duration of having the disease, type of therapy used for diabetes, history of diabetes in the first degree family, and any other major disease. In both phases of the study, for the completion of the questionnaires, the first author interviewed all patients face-to-face.

### 2.4. The Secondary Outcome Measures

In addition to our primary outcome measures, we also calculated BMI and means and standard deviations (SD) of the last three months' serum levels of HbA1c, FBS, cholesterol, and triglycerides (as described above) at baseline and also after three months.

### 2.5. Statistical Analysis

We used the Kolmogorov–Smirnov test to determine the normal distribution of the data: in the case of normal distribution, the analysis of variance was used to compare the difference in the sum scores between group pairs, and in the case of significant differences among the three groups, Tukey's test was used. In the case of nonnormal distribution of data, group pairs were compared using the nonparametric method of multiple comparisons. An alpha level of 0.05 was used for all statistical tests. All statistical analyses were performed using SPSS for Windows (version 21).

## 3. Results

### 3.1. Participants

**Figure 1:**
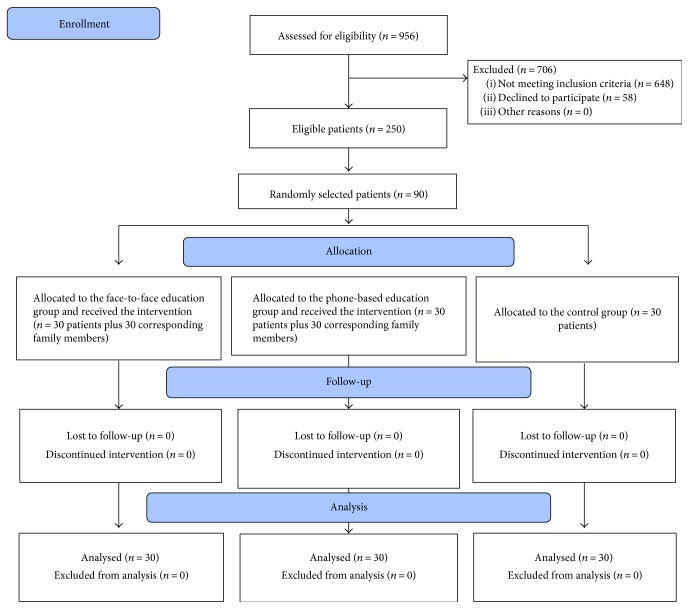
Flow chart of patient recruitment into the study.

During the study period, 90 patients were selected and randomized into three thirty-patient equal groups: the face-to-face family-oriented education group (group 1), the telephone-based family-oriented education group (group 2), and the control group (group 3).  Figure 1 shows the study's Consolidated Standards of Reporting Trials (CONSORT) flow diagram for patient recruitments. With regard to demographics, analysis showed no significant difference among the three study groups (Table 1). Most participants were male and on oral antidiabetic therapy.

### 3.2. Overall Mean Self-Care Scores

The overall mean score for each patient was calculated by summing SDSCA items' scores. The Kruskal-Wallis test showed no significant differences in overall mean self-care scores among the three groups at the beginning of the study. After the intervention, there were significant differences in mean and standard deviation of overall self-care scores among group 1 (100.82 ± 14.56), group 2 (92.93 ± 11.09), and group 3 (49.46 ± 16.35) (*P* = 0.0001) (Table 2). Comparison of the two intervention groups showed significantly higher overall scores in face-to-face education group than the telephone-based group (*P* = 0.011) (Table 3).

### 3.3. Self-Care Scores of Different SDSCA Domains

With regard to the mean dietary adherence scores, the results of the analysis of variance showed no significant differences among the three study groups in the preintervention period (*P* = 0.352). However, at the end of the study, there were significant differences among the three groups' mean dietary adherence scores (group 1: 30.5 ± 9.59, group 2: 25.9 ± 4.33, and group 3: 12.96 ± 6.91; *P* = 0.0001) (Table 2). The results of Tukey's test showed a significantly higher score in mean dietary adherence for the face-to-face group compared to the telephone-based group after the intervention (*P* = 0.043) (Table 4).

At the beginning of the study, the results of the Kruskal-Wallis test showed no significant differences among the three groups in mean scores of physical activity, blood glucose monitoring, foot care, and medication adherence. However, after the intervention, analysis showed a significant difference in mean physical activity scores of group 1 (10.73 ± 2.71), group 2 (9.33 ± 2.6), and group 3 (3.8 ± 3.18) (*P* = 0.0001) (Table 2). For the physical activity, the rank sum test showed significantly higher scores in the face-to-face group than in the telephone-based group (*P* = 0.04) (Table 4).

After the intervention, mean blood glucose monitoring scores of groups 1, 2, and 3 were 8.63 ± 3.46, 8.66 ± 2.96, and 1 ± 1.74, respectively (*P* = 0.0001). Further analysis showed that after the intervention, the scores of the two intervention groups were not statistically different (Table 2).

Mean foot care scores were 29.93 ± 5.28, 28.06 ± 5.73, and 11.23 ± 8.57 in groups 1, 2, and 3, respectively (*P* = 0.0001), with no difference between the two intervention groups after the intervention. However, regarding mean medication adherence scores, there were no significant differences among the three study groups neither before nor after the intervention (Table 2).

### 3.4. Clinical Outcomes

The results of the analysis of variance showed that in the beginning of the study, means and SDs of BMI, HbA1c, FBS, cholesterol, and triglyceride were not different among the three study groups. However, the interventions improved means and SDs of TG (132.7 ± 55.32 mg/dl in the face-to-face group and 118.7 ± 54.81 mg/dl in the telephone-based group versus 166.63 ± 53.94 mg/dl in the control group after the intervention; *P* = 0.003) (Table 3). Similar results were observed for cholesterol values (154.53 ± 39.4 mg/dl for the face-to-face group and 148.53 ± 49.4 mg/dl for the telephone-based intervention group versus 180.23 ± 49.9 mg/dl for the control group; *P* = 0.02). Further analysis showed that this significant difference was mainly associated with the telephone-based group (telephone-based versus control: *P* = 0.026; face-to-face versus control: *P* = 0.088) (Table 4). With regard to FBS and HbA1c, despite the decreasing trend in the intervention groups, this change did not reach statistical significance (Table 3).

## 4. Discussion

Our study aimed to compare the effects of a family-oriented education, delivered via different methods face-to-face and telephone-based methods, on SDSCA scores and clinical outcomes. Our results showed higher self-care scores, in the total score, mean dietary adherence score, and physical activity score as well as in lipid profiles in the intervention groups compared to the control group. Regarding mean dietary adherence and physical activity, comparison between the two intervention groups revealed better results in the face-to-face education group compared to the telephone-based education group. However, the improvements in blood glucose monitoring behavior and foot care were comparable between the two intervention groups. Even, the telephone-based group had better controlled cholesterol level compared to the face-to-face group. Given the fact that the telephone-based group had generally better outcomes compared to the control group, this finding indicates the potential value of telephone in diabetes education. Moreover, in our study, the medication adherence scores did not improve in any of intervention groups. The reason for this similar medication adherence could be due to two facts: first, the medication adherence score was high in all the three study groups before the intervention (>20 out of 21 score), and second, the study population envisioned medication as the most effective treatment for their disease and took their medication regimens rather seriously.

The researchers searched for few studies that have evaluated patients' self-care behavior by SDSCA in order to compare our results with theirs. Sacco et al. investigated the effects of a regular telephone-based intervention for 6 months and reported that it could improve dietary and exercise adherence in patients with T2DM [25]. Their results are in line with ours. Dietary adherence and regular physical activity are two cornerstones of lifestyle modifications in diabetes [26]. In this regard, previous studies have shown that family support could improve self-sufficiency and thereby increase patients' dietary adherence and physical condition monitoring [27]. This confirms the results of our study. In fact, the efficacy and feasibility of family-oriented intervention in our study to improve physical activity indicate that inclusion of educating patients and their family members in diabetes care can be an effective strategy to achieve better outcome in diabetes.

Studies have noted glycemic control as the primary goal of diabetic control. Therefore, we also investigated HbA1c and FBS levels before and after the study; however, no significant change was shown among the three groups. Different results have been reported by previous studies investigating the effect of different behavioral therapies on patients with T2DM. Researchers comparing face-to-face and telephone-based education (without family members) have shown similar weight loss and glucose control in both groups [28]. We found similar result by including family members in the educational program. Researchers providing face-to-face education for families (during clinic visits, family meetings, and home visits) have established favorable results on diabetic control after 12 months [18]. Another study also reported increased knowledge and a decreased HbA1c in the intervention group after one month [20]. However, in our study, the decrease in HbA1c and FBS was not statistically significant after 3 months. In line with the results of the present study, the study by Krein et al. showed little difference in HbA1c after a telephone-based intervention during an 18-month intervention. These researchers associated this finding with several patient-related and organizational factors [29]. Regarding these controversial findings in the literature, we suggest that future studies consider evaluating this outcome after similar interventions.

As diabetic patients face various complications during diabetes' prolonged course, educating patients on proper management and prevention of complications is of great importance. Thus, foot care, aimed to prevent diabetic foot, was another aspect of self-care dealt with in the present study. There was a significant improvement in foot care behavior with both face-to-face and telephone-based family-oriented interventions. Similar finding has been reported in a study focusing on face-to-face family-oriented interventions [20].

As an important factor for cardiovascular complications in diabetes, we also evaluated the change in the lipid profile of patients. We found a significant improvement in TG levels in both intervention groups and in cholesterol levels in the telephone-based group. This is in accordance with the results of other studies. For example, the study by Nuti et al. indicated that cholesterol levels decreased in patients with T2DM after four 90–120-minute teaching classes, held weekly, with family members [30]. Another study showed similar results in triglyceride levels after eight months of self-management education [31]. Even one study reported a significant decrease in both triglyceride and cholesterol levels after 6 months [32]. Therefore, these favorable results indicate that family-oriented education can be an effective measure in decreasing the lipid profile of diabetic patients.

Despite several strengths of the present study, it has also some limitations: this study was conducted over a relatively short period of three months. Further studies are required to investigate whether or not these early beneficial effects would last over a longer term. Another limitation of the present study was the relatively small number of patients. Future studies are recommended to recruit larger sample sizes. The next limitation was our focus on the outcomes of the intervention in patients with T2DM. Therefore, our findings may not be generalizable to all diabetes patients. Future studies are needed to confirm the efficacy and feasibility of such family-oriented educational interventions on the outcomes of other diabetes patient populations. There was also a possibility of confounding variables, including noncontrollable variables, such as the psychomental characteristics of patients and the cultural background of patients and their families, as well as their motivations that could affect their learning function. The controllable variables, such as demographic characteristics, including age, gender, family history of diseases, marital status, residential status, and educational level, were controlled in the present study by matched randomized groups.

In conclusion, the present study showed the beneficiary effects of education of patients and their family members on total self-care scores and mean dietary adherence, physical activity, glucose monitoring, and foot care scores in the intervention groups compared to the control group. Despite the superiority of the face-to-face education method over the telephone-based education regarding dietary adherence and physical activity, the telephone-based intervention had comparable and even better results than the face-to-face education in outcomes like glucose monitoring, foot care, and cholesterol levels. It is noteworthy that the telephone-based education generally resulted in better outcomes compared to the control. This finding points out the potential value of low-cost telephone technology in diabetes education. In conclusion, our study findings highlight the value of including family-oriented education as a regular component of diabetes care.

## Figures and Tables

**Table 1:** Demographic characteristics of participants in the three study groups.

Variable		Control, *N* (%)	Face-to-face education group, *N* (%)	Phone education group, *N* (%)	Chi-square
Gender	Female	11 (36.7)	15 (50)	13 (43.3)	*P* = 0.604
	Male	19 (63.3)	15 (50)	17 (56.7)	

Marital status	Married	29 (96.7)	28 (93.3)	28 (93/3)	*P* = 0.761
	Single	1 (3.3)	2 (6.7)	2 (6.7)	

Employment status	Currently unemployed	16 (53.3)	14 (46.7)	17 (56/7)	*P* = 0.565
	Employed	9 (30)	10 (33.3)	9 (30)	
	Retired	5 (16.7)	6 (20)	4 (13.3)	

Family history of diabetes	Yes	19 (63.3)	12 (40)	15 (50)	*P* = 0.304
	No	11 (36.7)	18 (60)	15 (50)	

Education level	Primary and secondary school	14 (46.7)	15 (50)	11 (36.7)	*P* = 0.823
	High school & university	16 (53.3)	15 (50)	19 (63.3)	

Treatment of diabetes	Insulin	2 (6.7)	3 (10)	7 (23.3)	*P* = 0.283
	Oral therapy	19 (63.3)	21 (70)	18 (60)	
	Both oral therapy & insulin	9 (30)	6 (20)	5 (16.7)	

Age mean (SD)		50.6 (3.74)	49.9 (4.98)	49.46 (4.76)	*P* = 0.798^∗^
Disease duration mean (SD)		11.33 (5.66)	10.33 (6.89)	9.26 (5.47)	*P* = 0.38^∗^

^∗^Tested by analysis of variance.

**Table 2:** Comparison of self-care and its domains among the three groups at the beginning and at the end of the study.

Variable		Control		Face-to-face education		Phone-based education		*P* value
		Mean ± SD	Mean ranks	Mean ± SD	Mean ranks	Mean ± SD	Mean ranks	
Dietary adherence (0–35)	Preintervention	15.06 ± 7.03	—	12.7 ± 5.71	—	13.5 ± 6.13	—	*F*_(2,87)_ = 1.056 *P* = 0.352^∗∗^
	Postintervention	12.96 ± 6.91	—	30.5 ± 9.59	—	25.9 ± 4.33	—	*F*_(2,87)_ = 46.91 *P* = 0.0001^∗∗^

Physical activity (0–14)	Preintervention	5.9 ± 3.55	52.63	4.9 ± 3.68	42.33	4.53 ± 3.03	41.53	*χ* ^2^ = 3.44 df = 2 *P* = 0.179^∗^
	Postintervention	3.8 ± 3.18	19.88	10.73 ± 2.71	63.22	9.33 ± 2.6	53.4	*χ* ^2^ = 46.17 df = 2 *P* = 0.0001^∗^

Blood glucose monitoring (0–14)	Preintervention	3.97 ± 2.7	47.12	3.58 ± 1.8	41.45	3.47 ± 2.56	47.93	*χ* ^2^ = 1.28 df = 2 *P* = 0.526^∗^
	Postintervention	1.74 ± 1	16.67	8.63 ± 3.46	59.82	8.66 ± 2.96	60.02	*χ* ^2^ = 55.78 df = 2 *P* = 0.0001^∗^

Foot care (0–35)	Preintervention	14.3 ± 9.07	50.52	19.13 ± 6.86	33.47	15.16 ± 8.96	52.52	*χ* ^2^ = 2.66 df = 2 *P* = 0.098^∗^
	Postintervention	11.23 ± 8.57	19.45	29.93 ± 5.28	61.55	28.06 ± 5.73	55.5	*χ* ^2^ = 46.02 df = 2 *P* = 0.0001^∗^

Medication adherence (0–21)	Preintervention	20.86 ± 0.73	48.42	20.83 ± 0.74	47.05	20.36 ± 1.42	41.03	*χ* ^2^ = 5.006 df = 2 *P* = 0.082^∗^
	Postintervention	20.46 ± 2.92	44.98	21 ± 0.001	46.5	20.93 ± 0.365	45.02	*χ* ^2^ = 1.01 df = 2 *P* = 0.603^∗^

The overall self-care (0–119)	Preintervention	58.83 ± 18.95	51.4	49.36 ± 15.74	36.73	56.13 ± 17.64	48.38	*χ* ^2^ = 5.29 df = 2 *P* = 0.07^∗^
	Postintervention	49.46 ± 16.35	16.9	100.82 ± 14.56	65.78	92.93 ± 11.09	53.82	*χ* ^2^ = 57.1 df = 2 *P* = 0.001^∗^

^∗∗^Analysis of variance. ^∗^Kruskal-Wallis.

**Table 3:** Pairwise comparison of self-care and its domains as well as clinical outcomes among the three groups at the beginning and at the end of the study.

Variable	Group	Control	Face-to-face education
Dietary adherence	Face-to-face education	*P* = 0.0001^∗∗^	—
	Phone education	*P* = 0.0001^∗∗^	*P* = 0.043^∗∗^

Physical activity	Face-to-face education	*P* = 0.0001^∗^	—
	Phone education	*P* = 0.0001^∗^	*P* = 0.04^∗^

Blood glucose monitoring	Face-to-face education	*P* = 0.0001^∗^	—
	Phone education	*P* = 0.0001^∗^	*P* = 0.994^∗^

Foot care	Face-to-face education	*P* = 0.0001^∗^	—
	Phone education	*P* = 0.0001^∗^	*P* = 0.235^∗^

The overall self-care	Face-to-face education	*P* = 0.0001^∗^	—
	Phone education	*P* = 0.0001^∗^	*P* = 0.011^∗^

BMI	Face-to-face education	*P* = 0.836^∗^	—
	Phone education	*P* = 0.827^∗^	*P* = 0.482^∗^

FBS (mg/dl)	Face-to-face education	*P* = 0.384^∗^	—
	Phone education	*P* = 0.766^∗^	*P* = 0.804^∗^

Cholesterol (mg/dl)	Face-to-face education	*P* = 0.088^∗^	—
	Phone education	*P* = 0.026^∗^	*P* = 0.872^∗^

Triglyceride (mg/dl)	Face-to-face education	*P* = 0.048^∗^	—
	Phone education	*P* = 0.003^∗^	*P* = 0.584^∗^

HbA1c (%)	Face-to-face education	*P* = 0.344^∗^	—
	Phone education	*P* = 0.236^∗^	*P* = 0.971^∗^

^∗∗^Tukey's test. ^∗^The rank sum difference.

**Table 4:** Comparison of patient clinical outcomes among the three groups at the beginning and at the end of the study.

Variable		Control group (mean ± SD)	Phone group (mean ± SD)	Face-to-face group (mean ± SD)	ANOVA
BMI	Preintervention	3.85 ± 28.25	4.06 ± 29.41	4.77 ± 28.27	*F*_(2, 87)_ = 0.72
					*P* = 0.48
	Postintervention	3.64 ± 28.71	3.99 ± 29.31	4.67 ± 28.08	*F*_(2, 87)_ = 0.67
					*P* = 0.51

FBS (mg/dl)	Preintervention	42.56 ± 147.3	46.7 ± 154.5	50.63 ± 163.76	*F*_(2, 87)_ = 0.93
					*P* = 0.39
	Postintervention	38.69 ± 150.9	33.43 ± 138.33	37.65 ± 144.3	*F*_(2, 87)_ = 0.88
					*P* = 0.41

Cholesterol (mg/dl)	Preintervention	48.76 ± 167.7	49.52 ± 173.7	52.46 ± 172.5	*F*_(2, 87)_ = 0.11
					*P* = 0.88
	Postintervention	49.9 ± 180.23	39.4 ± 154.53	49.4 ± 148.53	*F*_(2, 87)_ = 3.93
					*P* = 0.02

Triglyceride (mg/dl)	Preintervention	53.36 ± 162.83	96.12 ± 163.46	69.11 ± 133.9	*F*_(2, 87)_ = 1.52
					*P* = 0.22
	Postintervention	53.94 ± 166.63	55.32 ± 132.7	54.81 ± 118.7	*F*_(2, 87)_ = 6.09
					*P* = 0.003

HbA1c (%)	Preintervention	1.7 ± 7.8	1.1 ± 8.2	1.5 ± 7.9	*F*_(2, 87)_ = 0.65
					*P* = 0.52
	Postintervention	1.5 ± 7.8	1.2 ± 7.3	1.2 ± 7.2	*F*_(2, 87)_ = 1.56
					*P* = 0.21
